# Supplementary material for: GC Content Across Insect Genomes: Phylogenetic Patterns, Causes and Consequences
Source: J Mol Evol. 2024 Mar 15;92(2):138–52. doi: 10.1007/s00239-024-10160-5 (PMC10978632; doi:10.1007/s00239-024-10160-5)
Supplement: Supplementary file 1 — Supplementary file1 (DOCX 1444 KB) [file 239_2024_10160_MOESM1_ESM.docx]

Supplementary Materials

GC content across insect genomes:

phylogenetic patterns, causes and consequences

Riccardo G. Kyriacou^1^, Peter O. Mulhair^1^, Peter W. H. Holland^1*^

*^1^Department of Biology, University of Oxford, 11a Mansfield Road, Oxford OX1 3SZ, UK*

**Corresponding author:* [*peter.holland@biology.ox.ac.uk*](mailto:peter.holland@biology.ox.ac.uk)

##

**Supplementary Figures**

Supplementary Figure S1: Labelled species trees for Lepidoptera, Diptera, Hymenoptera and Coleoptera

Supplementary Figure S2: Chromosome sizes for Lepidoptera, Diptera, Hymenoptera and Coleoptera

Supplementary Figure S3: Amino acid trees for genes encoding OGG1, TDG, SMUG1

Supplementary Figure S4: Codon usage against tRNA correlations, outliers removed

Supplementary Figure S5: tRNA gene count and anticodon GC% across Diptera

**Supplementary Tables**

Supplementary Table S1: GC mean values, two-way ANOVA, and TukeyHSD post-hoc test on GC content with DNA type and order

Supplementary Table S2: Table S2: Marginal likelihood outputs from BayesTraits and calculation of Log Bayes Factor for each insect order

Supplementary Table S3: GC content against distance from telomere regression analysis

Supplementary Table S4: tRNA against codon usage analysis

Supplementary Table S5: Sneath value analysis

##

## **Supplementary Figures**

### Supplementary Figure S1: Labelled species trees for Lepidoptera, Diptera, Hymenoptera and Coleoptera

### **
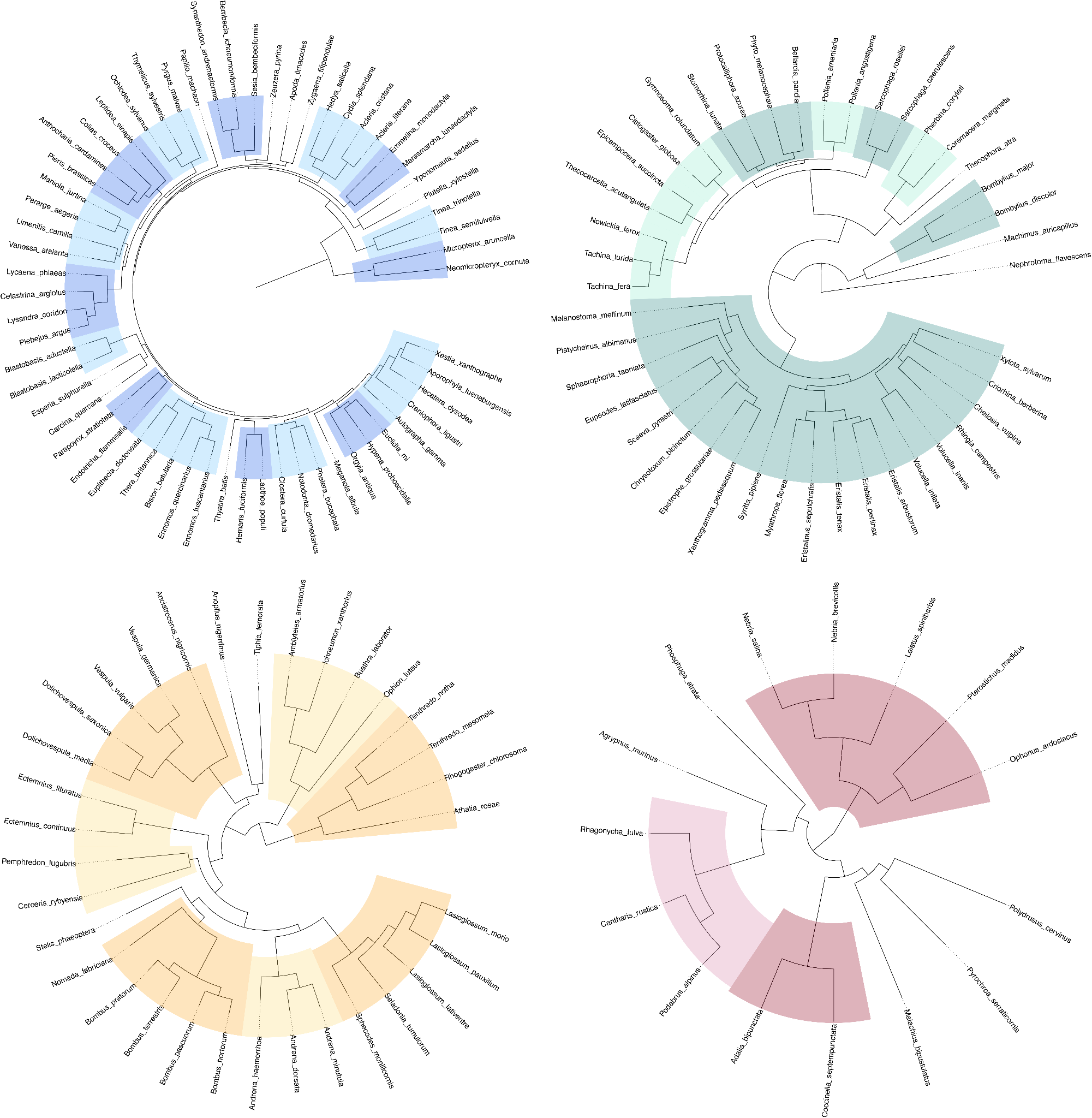
**

**Supplementary Figure S1. Radial species trees for 150 insects in this study. 60 Lepidoptera (top left), 42 Diptera (top right), 33 Hymenoptera (bottom left), 15 Coleoptera (bottom right). Species trees generated from in OrthoFinder using default settings and STAG methodology.**

### Supplementary Figure S2: Chromosome sizes for Lepidoptera, Diptera, Hymenoptera and Coleoptera

**
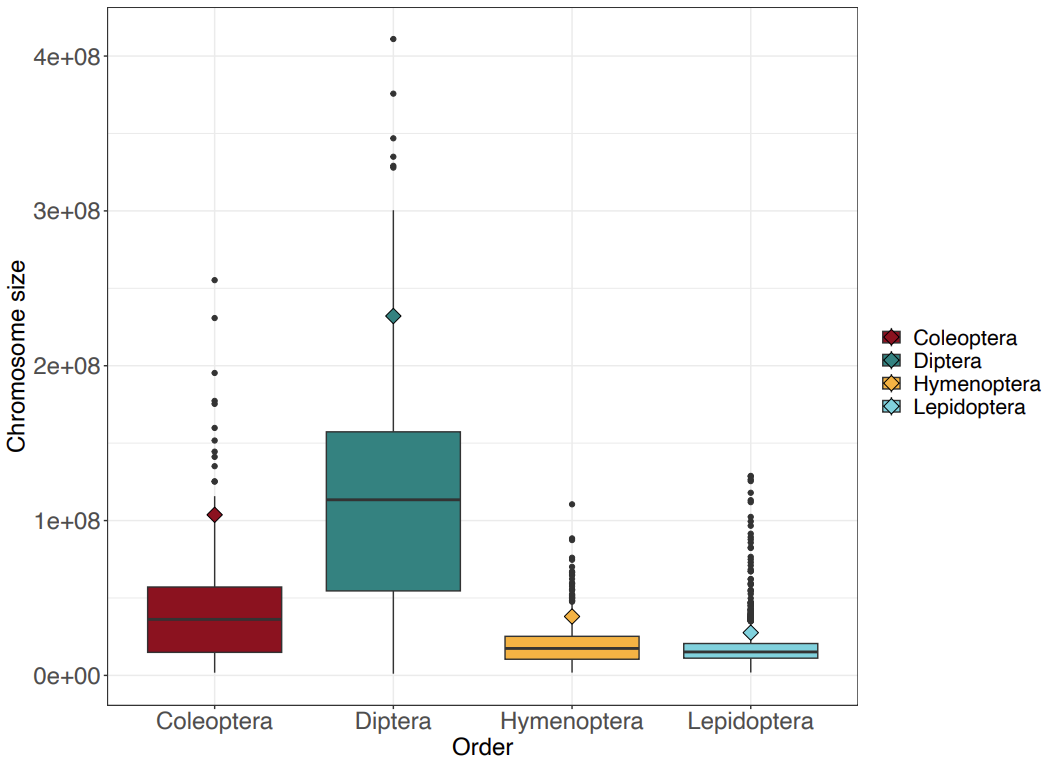
**

**Supplementary Figure S2. Comparison of chromosome sizes for Coleoptera (red), Diptera (green), Hymenoptera (yellow) and Lepidoptera (light blue) chromosomes. Each chromosome for each species plotted individually for 60 Lepidoptera (1827 chromosomes), 42 Diptera (237 chromosomes), 33 Hymenoptera (446 chromosomes) and 15 Coleoptera (197 chromosomes) species. Horizontal bars are raw means of the data points; diamonds indicate phylogenetically informed mean values. Box values correspond to the first and third quartiles. Upper whisker extends to the largest value 1.5x interquartile range or distance between the first and third quartiles. Lower whisker extends to the smallest value 1.5 * IQR. Any data points beyond these limits are represented as outlier points.**

##

### Supplementary Figure S3: Amino acid trees for genes encoding OGG1, TDG, SMUG1

###
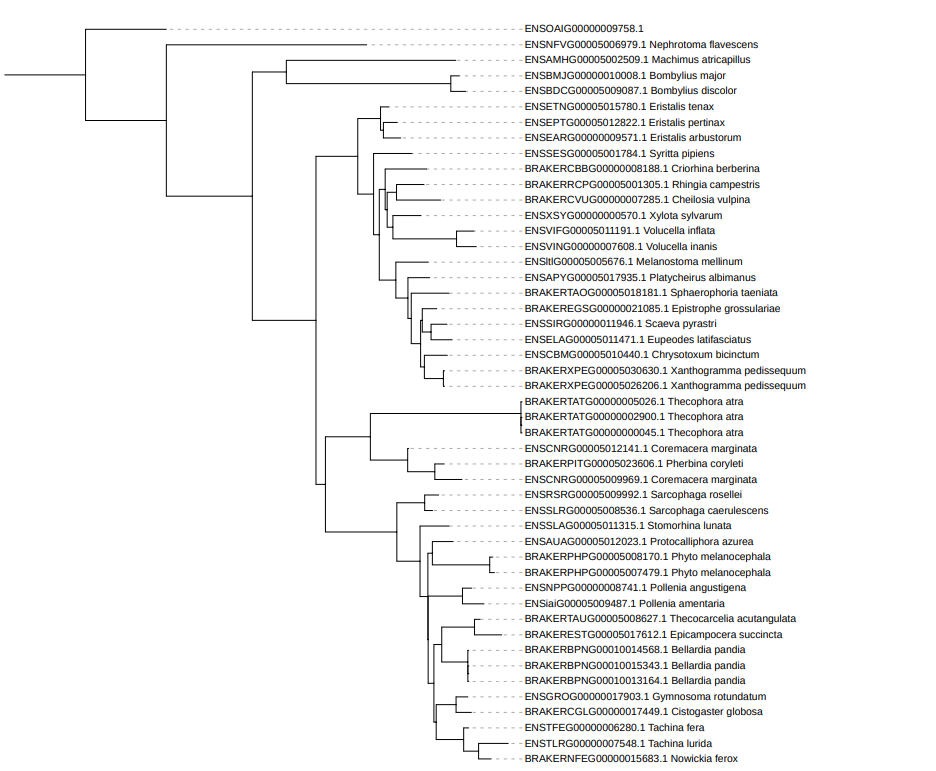

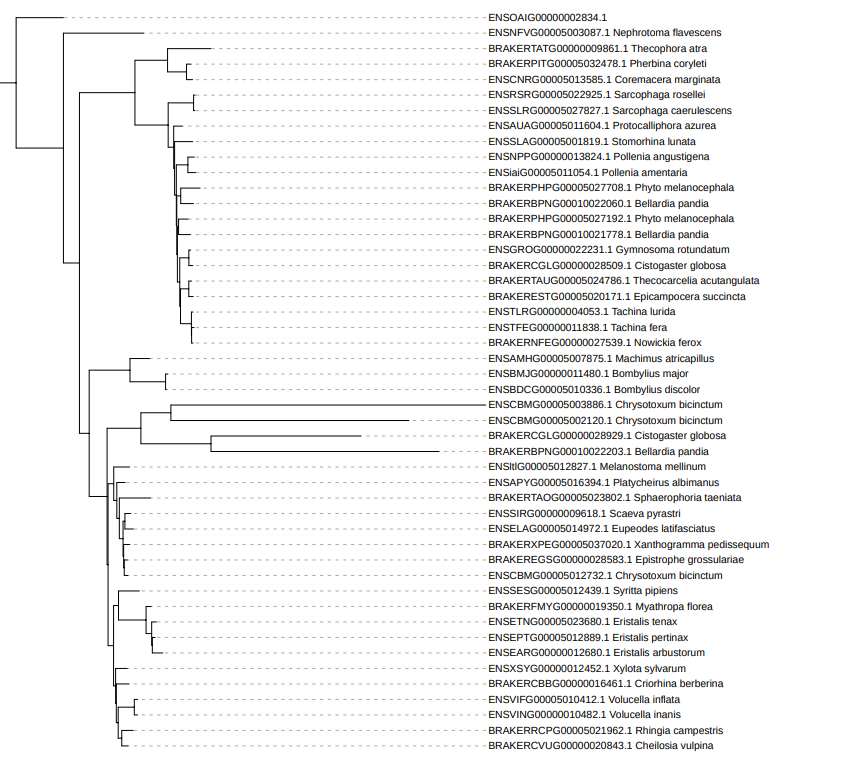


OGG1

###

###

###

TDG


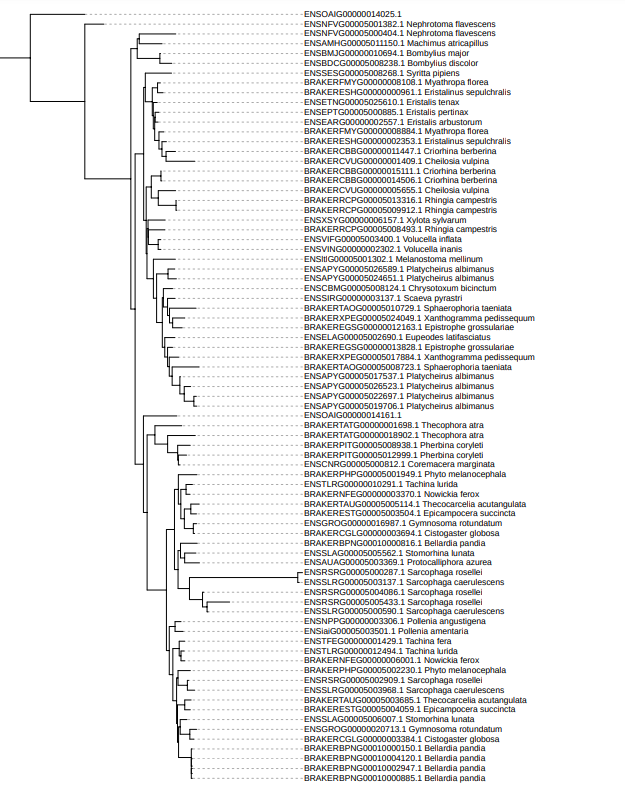


SMUG1

**Supplementary Figure S3 - Gene trees built from amino acid sequences for the Base Excision Repair (BER) genes *ogg1* (top)*, tdg* (middle) and *smug1* (bottom) found across dipteran species (plus one outgroup species, *Orgyia antiqua* Lepidoptera). The OGG1 protein primarily targets oxidizes G resides which can otherwise lead to G:C to A:T mutations; TDG primarily targets T:G pairs resultant from deamination of 5methyl-C residues, but also can target U:G pairs; SMUG1 primarily targets U:G pairs generated by deamination of C residues. Tree tip labels show gene ID followed by species names. Gene trees built using IQTREE.**

### Supplementary Figure S4: Codon usage against tRNA correlations with outliers removed

###
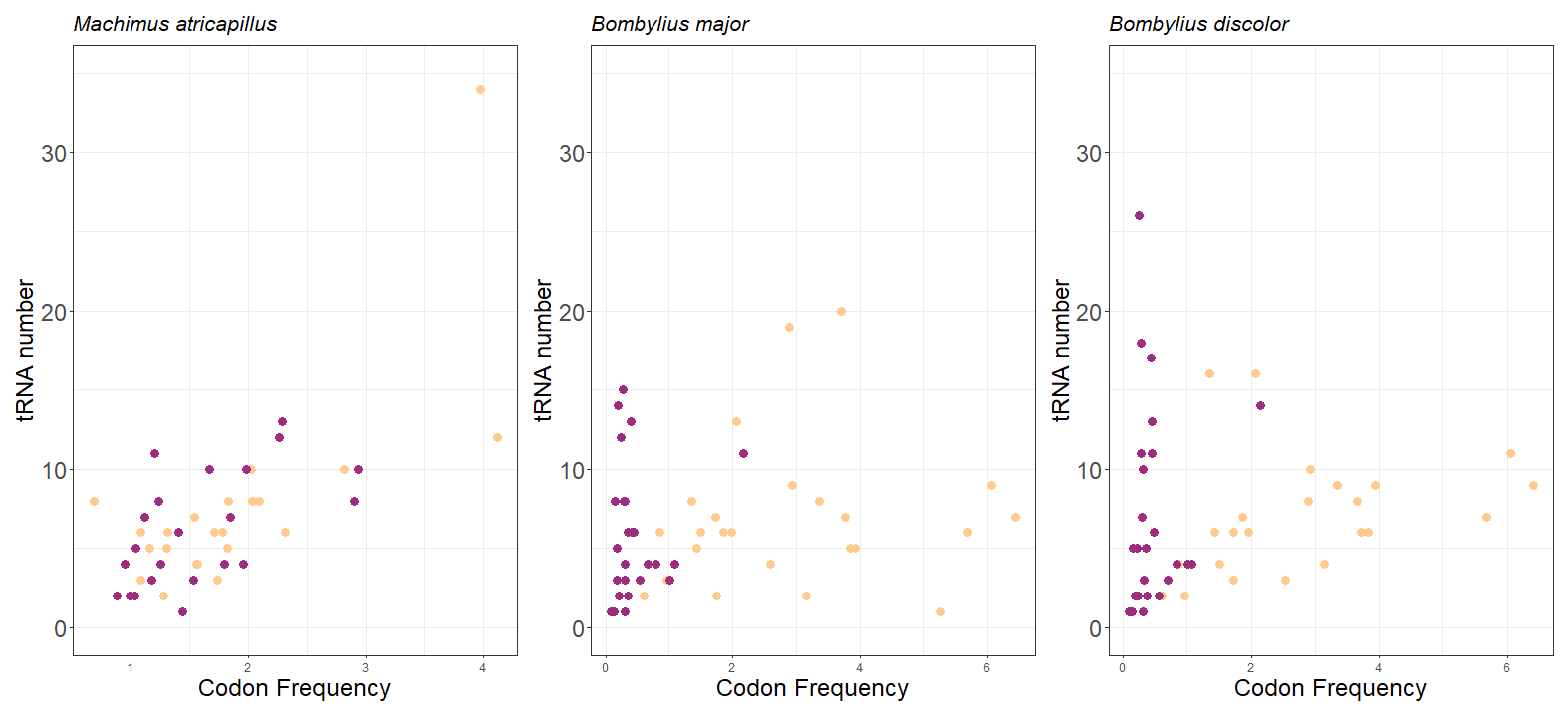


##

##

##

##

##

##

**Supplementary Figure S4 - Plots of tRNA gene counts against codon frequency in Dipteran SCOs. Outliers in *B. major* and *B. discolor* (that is tRNA genes with anticodons binding Ala-GCC, Phe-TTC, and Ser-TCC) have been excluded from the data, in order to better understand the relationship between the two variables.**

### Supplementary Figure S5: tRNA gene count and anticodon GC% across Diptera

###
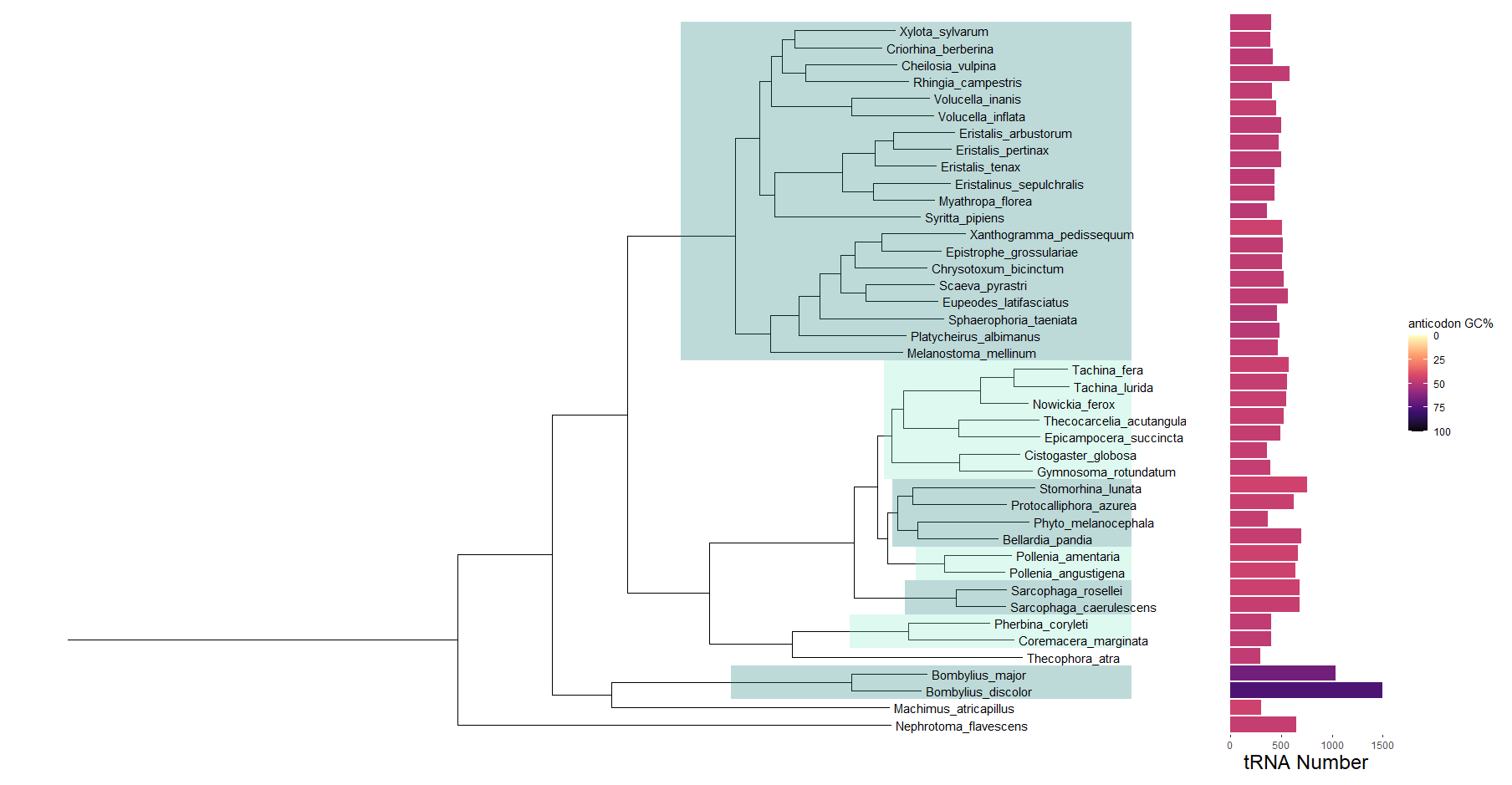


**Supplementary Figure S5 - Dipteran species tree plotted next to bar chart showing tRNA gene number for each species. tRNA numbers predicted from dipteran genomes using tRNAscan-SE 2.0. For each species, bars are coloured according to the average GC content of the tRNA anticodon. *Bombylius major* and *Bombylius discolor* are outliers in both tRNA number and anti-codon GC% (*B. major*: 1033, 66.9%; *B. discolor*: 1493, 75.7%).**

## **Supplementary Tables**

### Table S1: GC means, Two-way ANOVA, and TukeyHSD post-hoc test on GC content with DNA type and order

**Table S1 (a) Phylogenetically informed mean GC% (the estimated mean of the clade accounting for phylogeny) and sample genome mean GC% (uncorrected mean for the samples ignoring phylogeny) per order:**

| **Phylogenetically informed & genome GC mean** | | | | |
| --- | --- | --- | --- | --- |
| **Order** | **Data type** | **Phylogenetically informed mean** | **Sample genome mean** | **Sample size** |
| Lepidoptera | Genome GC | 37.18409 | 36.75300 | 60 |
| Diptera | Genome GC | 33.32352 | 34.31024 | 42 |
| Hymenoptera | Genome GC | **40.70223** | 40.21364 | 33 |
| Coleoptera | Genome GC | 33.37498 | 33.16667 | 15 |
| Lepidoptera | CDS GC | **47.79085** | 48.17183 | 60 |
| Diptera | CDS GC | 39.40374 | 40.78143 | 42 |
| Hymenoptera | CDS GC | 45.92266 | 44.99667 | 33 |
| Coleoptera | CDS GC | 40.94129 | 40.51933 | 15 |
| Lepidoptera | SCO GC3 | **50.8443** | 49.75517 | 60 |
| Diptera | SCO GC3 | 34.24349 | 38.24048 | 42 |
| Hymenoptera | SCO GC3 | 44.42901 | 41.98212 | 33 |
| Coleoptera | SCO GC3 | 37.0399 | 36.66733 | 15 |

**Table S1 (b) Two-way ANOVA test using phylogenetically informed GC% means:**

|  | **Df** | **Sum Sq** | **Mean Sq** | **F value** | **Pr(>F)** |
| --- | --- | --- | --- | --- | --- |
| **DNA_type** | 2 | 117.31 | 58.66 | 7.029 | 0.0268 * |
| **Order** | 3 | 203.38 | 67.79 | 8.124 | 0.0156 * |
| **Residuals** | 6 | 50.07 | 8.35 |  |  |

Significance codes: * 0.05

**Table S1 (c) Tukey HSD test:**

| **$DNA_type** |  |  |  |  |
| --- | --- | --- | --- | --- |
|  | **diff** | **lwr** | **upr** | **p adj** |
| **GC_genome-GC_cds** | -7.36843 | -13.6359189 | -1.100941 | 0.0261900 |
| **GC3-GC_cds** | -1.87546 | -8.1429489 | 4.392029 | 0.6496791 |
| **GC3-GC_genome** | 5.49297 | -0.7745189 | 11.760459 | 0.0802893 |

| **$Order** |  |  |  |  |
| --- | --- | --- | --- | --- |
|  | **diff** | **lwr** | **upr** | **p adj** |
| **Diptera-Coleoptera** | -1.461807 | -9.62687071 | 6.703257 | 0.9220742 |
| **Hymenoptera-Coleoptera** | 6.565910 | -1.59915404 | 14.730974 | 0.1115410 |
| **Lepidoptera-Coleoptera** | 8.154357 | -0.01070738 | 16.319421 | 0.0502626 |
| **Hymenoptera-Diptera** | 8.027717 | -0.13734738 | 16.192781 | 0.0534842 |
| **Lepidoptera-Diptera** | 9.616163 | 1.45109929 | 17.781227 | 0.0251166 |
| **Lepidoptera-Hymenoptera** | 1.588447 | -6.57661738 | 9.753511 | 0.9034581 |

**Table S1 (d) Levene's Test for Homogeneity of Variance and normality checks**

Top set of graphs is for tests applied to raw data, lower set of graphs is for phylogenetically-informed means.

|  | Df | F value | Pr(>F) |  |
| --- | --- | --- | --- | --- |
| group | 11 | 11.986 | < 2.2e-16 | *** |
|  | 438 |  |  |  |

**
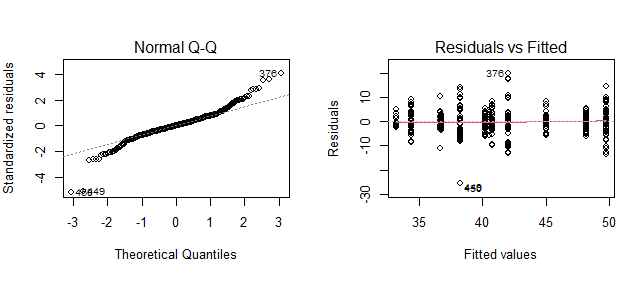

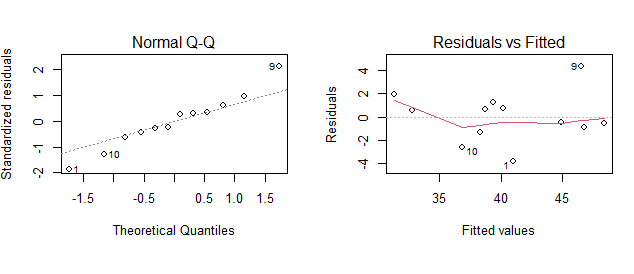
**

### Table S2: Marginal likelihood outputs from BayesTraits and calculation of Log Bayes Factor for each insect order

| **BayesTraits output from running DistData command (where M1 is complex model and M0 is model where no correlation is assumed)** |
| --- |
| *Lepidoptera M1 likelihood:* -158.059459  *Lepidoptera M0 likelihood:* -156.602127  *Log Bayes Factor* = 2(M1 – M0) = 2(-158.059459 - -156.602127) = -2.914664 |
| *Diptera M1:* -136.183732  *Diptera M0:* -138.174794  *Log Bayes Factor* = 2(-136.183732- -138.174794) = 3.9821239 |
| *Hymenoptera M1:* -112.619703  *Hymenoptera M0:* -112.299289  *Log Bayes Factor* = 2(-112.619703- -112.299289) = -0.6408279 |
| *Coleoptera M1:* -62.502342  *Coleoptera M0:* -62.306403  *Log Bayes Factor* = 2(-62.502342- -62.306403) = -0.3918779 |

### Table S3: GC content against distance from telomere regression analysis

**3a) Lepidoptera**

regression <- lm(GC3 ~ as.numeric(t_distance), data = mean_distance)

Signif. codes: 0 ‘***’ 0.001 ‘**’ 0.01 ‘*’ 0.05 ‘.’ 0.1 ‘ ’ 1

| Coefficients: |  |  |  |  |
| --- | --- | --- | --- | --- |
|  | Estimate | Std. Error | t value | Pr(>\|t\|) |
| (Intercept) | 5.483e+01 | 9.862e-01 | 55.603 | < 2e-16 *** |
| (t_distance) | -6.160e-07 | 1.437e-07 | -4.286 | 2.05e-05 *** |

Residual standard error: 9.143 on 769 degrees of freedom

Multiple R-squared: 0.02333, Adjusted R-squared: 0.02206

F-statistic: 18.37 on 1 and 769 DF, p-value: 2.053e-05

**3b) Coleoptera**

| Coefficients: |  |  |  |  |
| --- | --- | --- | --- | --- |
|  | Estimate | Std. Error | t value | Pr(>\|t\|) |
| (Intercept) | 4.079e+01 | 5.780e-01 | 70.57 | < 2e-16 *** |
| (t_distance) | -2.771e-07 | 4.075e-08 | -6.80 | 1.58e-11 *** |

Residual standard error: 6.187 on 1327 degrees of freedom

Multiple R-squared: 0.03367, Adjusted R-squared: 0.03294

F-statistic: 46.24 on 1 and 1327 DF, p-value: 1.579e-11

**3c) Hymenoptera**

| Coefficients: |  |  |  |  |
| --- | --- | --- | --- | --- |
|  | Estimate | Std. Error | t value | Pr(>\|t\|) |
| (Intercept) | 4.863e+01 | 5.768e-01 | 84.304 | <2e-16 *** |
| (t_distance) | -1.213e-07 | 7.234e-08 | -1.676 | 0.0939 |

Residual standard error: 7.121 on 1941 degrees of freedom

Multiple R-squared: 0.001445, Adjusted R-squared: 0.000931

F-statistic: 2.81 on 1 and 1941 DF, p-value: 0.09385

**3b) Diptera**

| Coefficients: |  |  |  |  |
| --- | --- | --- | --- | --- |
|  | Estimate | Std. Error | t value | Pr(>\|t\|) |
| (Intercept) | 3.513e+01 | 3.681e-01 | 95.41 | < 2e-16 *** |
| (t_distance) | -2.546e-08 | 9.718e-09 | -2.62 | 0.00895 ** |

Residual standard error: 4.281 on 846 degrees of freedom

Multiple R-squared: 0.008049, Adjusted R-squared: 0.006876

F-statistic: 6.864 on 1 and 846 DF, p-value: 0.008951

### Table S4: tRNA against codon usage analysis

3a) *Bombylius major*

Pearson's product-moment correlation

data: trna_codon$tRNA_count and trna_codon$abs_freq

**t = -0.94885, df = 50, p-value = 0.3473**

alternative hypothesis: true correlation is not equal to 0

95 percent confidence interval:

**-0.3916799 0.1451740**

sample estimates:

cor

**-0.1329954**

3b) *Bombylius discolor*

Pearson's product-moment correlation

data: trna_codon$tRNA_count and trna_codon$abs_freq

**t = -0.98025, df = 50, p-value = 0.3317**

alternative hypothesis: true correlation is not equal to 0

95 percent confidence interval:

**-0.3953982 0.1408642**

sample estimates:

cor

**-0.1373147**

3c) *Machimus atricapillus*

Pearson's product-moment correlation

data: trna_codon$tRNA_count and trna_codon$abs_freq

**t = -0.98025, df = 50, p-value = 0.3317**

alternative hypothesis: true correlation is not equal to 0

95 percent confidence interval:

**-0.3953982 0.1408642**

sample estimates:

cor

**-0.1373147**

### Table S4: Sneath value analysis

5a) Checking normality

Shapiro-Wilk normality test:

data: sneath_data$Bombylius_major

**W = 0.884, p-value < 2.2e-16**

data: sneath_data$Bombylius_discolor

**W = 0.9011, p-value < 2.2e-16**

data: sneath_data$Machimus_atricapillus

**W = 0.87683, p-value < 2.2e-16**

5b) *Bombylius majo*r vs *Bombylius discolor*

Pearson's product-moment correlation

data: sneath_data$Bombylius_major and sneath_data$Bombylius_discolor

**t = 86.776, df = 847, p-value < 2.2e-16**

alternative hypothesis: true correlation is not equal to 0

95 percent confidence interval:

**0.9408323 0.9544937**

sample estimates:

cor

**0.94809*8***

5b) *Bombylius majo*r vs *Machimus atricapillus*

Pearson's product-moment correlation

data: sneath_data$Bombylius_major and sneath_data$Machimus_atricapillus

**t = 29.102, df = 847, p-value < 2.2e-16**

alternative hypothesis: true correlation is not equal to 0

95 percent confidence interval:

**0.6717655 0.7392046**

sample estimates:

cor

**0.7070893**

5b) *Bombylius discolor* vs *Machimus atricapillus*

Pearson's product-moment correlation

data: sneath_data$Bombylius_discolor and sneath_data$Machimus_atricapillus

**t = 29.08, df = 847, p-value < 2.2e-16**

alternative hypothesis: true correlation is not equal to 0

95 percent confidence interval:

**0.6714693 0.7389597**

sample estimates:

cor

**0.7068193**
